# Effectiveness of different repetitive transcranial magnetic stimulation modalities on sleep function and depression in patients with post-stroke sleep disorders: a systematic review and network meta-analysis of randomized controlled trials

**DOI:** 10.3389/fneur.2025.1646383

**Published:** 2025-09-05

**Authors:** Chen Feng, Wei Zhang, Xiaoyang Cui, Peng Chen, Jing Wu, Juan Wang, Yangpu Zhang, Chanjuan Zheng

**Affiliations:** ^1^Hubei Provincial Hospital of Integrated Chinese and Western Medicine, Wuhan, China; ^2^Hubei Provincial Clinical Research Center for Stroke Rehabilitation of Integrated Traditional Chinese and Western Medicine, Wuhan, China; ^3^School of Exercise and Health, Shanghai University of Sport, Shanghai, China; ^4^Hubei University of Chinese Medicine, Wuhan, China

**Keywords:** repetitive transcranial magnetic stimulation, post-stroke sleep disorders, sleep function, depression, network meta-analysis

## Abstract

**Background:**

Post-stroke sleep disorders are common chronic complications that can severely impair patient recovery. Although post-stroke sleep disorders have been effectively treated using repetitive transcranial magnetic stimulation (rTMS), the relative efficacy of varied rTMS modalities remains unclear.

**Methods:**

We conducted a comprehensive search of the Cochrane Library, PubMed, Embase, Web of Science, Scopus, China National Knowledge Infrastructure, and Wanfang Data databases up to July 2024. Two investigators independently selected and analyzed the relevant studies, as well as evaluated the risk of bias, indirectness, and overall confidence in the network. A frequentist network meta-analysis was conducted to compare differences in the Pittsburgh Sleep Quality Index (PSQI) scores, sleep efficiency, and Hamilton Depression Scale (HAMD-17) scores following treatment with various rTMS modalities.

**Results:**

A total of 15 randomized controlled trials involving 1,113 patients with post-stroke sleep disorders were included. The rTMS protocols comprised low-frequency stimulation of the right dorsolateral prefrontal cortex (DLPFC), low-frequency stimulation of the bilateral DLPFC (b-DLPFC), and high-frequency stimulation of the left DLPFC. Compared with pharmacotherapy alone, low-frequency rTMS of the right DLPFC significantly improved PSQI scores, HAMD-17 scores, and sleep efficiency. Additionally, low-frequency rTMS of the b-DLPFC significantly improved PSQI scores and sleep efficiency compared with pharmacotherapy alone. In contrast, high-frequency rTMS of the left DLPFC showed no significant improvements in the PSQI scores, HAMD-17 scores, or sleep efficiency in comparison with pharmacotherapy alone. Moreover, no significant differences in efficacy were observed among the three rTMS modalities. Finally, probabilistic ranking suggested that low-frequency rTMS of the right DLPFC was optimal for enhancing PSQI scores, low-frequency rTMS of the b-DLPFC was most effective for improving sleep efficiency, and low-frequency rTMS of the right DLPFC was the most beneficial for reducing HAMD-17 scores.

**Conclusion:**

Low-frequency rTMS targeting the right DLPFC offers superior overall effectiveness in improving sleep function and alleviating depression in patients with post-stroke sleep disorders.

**Systematic review registration:**

https://www.crd.york.ac.uk/PROSPERO/view/CRD42024589437, identifier CRD42024589437.

## 1 Introduction

Stroke, also known as cerebrovascular accident, is an acute disease characterized by the rapid onset of localized brain function loss. According to the Global Burden of Disease Study 2019, stroke remains the second-leading cause of global mortality and the third-leading cause of disability-adjusted life years (DALYs) worldwide (1). Globally, stroke imposes an annual economic burden of approximately US$900 billion, encompassing both direct healthcare expenditures and indirect costs attributable to productivity losses from disability-adjusted life years (DALYs) and premature mortality (2). Post-stroke sleep disorders (PSSD), which include insomnia, sleep apnea, and circadian rhythm disturbances, are defined as disruptions in sleep quality, quantity, or timing following a stroke. These disorders may either emerge for the first time after the stroke or worsen if pre-existing, leading to clinically significant sleep disturbances that meet diagnostic criteria for sleep disorders (3). Furthermore, PSSD is a prominent issue during stroke recovery, with up to 75% of patients experiencing sleep-related problems after a stroke (4).

PSSD not only reduces the quality of life but also hinders post-stroke recovery. It is associated with higher rates of functional decline, long-term disability, and an increased risk of stroke recurrence (5). This post-stroke condition has also been found to be associated with prolonged hospitalization, impaired rehabilitation outcomes, and an elevated risk of long-term disability (6, 7). Additionally, patients with PSSD are at an increased risk of developing post-stroke depression (PSD), with depression affecting about one-third of these individuals (8, 9). PSD is a mood disorder characterized by persistent depressive symptoms and a loss of interest following a stroke. In severe cases, it may also involve difficulties with concentration, reduced self-esteem, and even suicidal ideation (10). PSD can substantially complicate the rehabilitation process by diminishing motivation, treatment adherence, and emotional well-being, ultimately worsening outcomes and delaying recovery (11). Moreover, depression and sleep have a bidirectional relationship, where poor sleep quality exacerbates depressive symptoms, while untreated depression escalates sleep problems. This vicious cycle between depression and sleep severely hinders the post-stroke recovery process (12, 13).

The importance of addressing sleep disorders and depression is underscored by several studies that have established a clear link between these conditions and worsened stroke recovery outcomes (8, 9). However, research on this topic has primarily focused on motor function recovery in patients with stroke, with relatively scarce clinical evidence on the management of sleep disorders and depression (11, 14). Given the significant impact of PSSD on recovery, novel treatment strategies, such as repetitive transcranial magnetic stimulation (rTMS), have gained attention as potential solutions. rTMS is a non-invasive brain stimulation technique that applies magnetic pulses to specific brain regions to modulate neural activity and enhance neuroplasticity (15). Recent studies have also highlighted the potential of rTMS in improving sleep quality (16). rTMS modulates cortical activity, promotes neuroplasticity, and improves sleep quality in stroke patients by reducing cortical hyperexcitability (17). Additionally, rTMS has been shown to effectively treat PSD by targeting the left dorsolateral prefrontal cortex (l-DLPFC), a brain region crucial for mood regulation and exhibiting reduced activity in individuals with depression (18).

Although rTMS has demonstrated positive effects on sleep quality and depression in patients with stroke, its role in managing PSSD requires further exploration. However, the efficacy of rTMS can vary depending on treatment duration, patient characteristics, and the specific rTMS protocol used, highlighting the need for further studies to optimize treatment strategies. Current rTMS protocols include high-frequency stimulation of the l-DLPFC, low-frequency stimulation of the bilateral DLPFC (b-DLPFC), and low-frequency stimulation of the right DLPFC (r-DLPFC) (19). However, additional research is necessary to determine the optimal rTMS protocol for treating patients with PSSD (20–22). This study aims to evaluate the comparative efficacy of different rTMS protocols in treating PSSD. A network meta-analysis is the appropriate method as it allows for the comparison of multiple treatments across various studies, providing a more comprehensive understanding of the relative effectiveness of each protocol (23).

## 2 Materials and methods

### 2.1 Selection criteria

A total of nine databases were searched for studies published in Chinese or English from their inception until July 31, 2024. These databases included the Cochrane Library, PubMed, Embase, Web of Science, Scopus, China National Knowledge Infrastructure (CNKI), and Wanfang Data. Within these databases, we will conduct a thorough search for gray literature and trial registries to ensure comprehensive coverage of all relevant studies. The retrieval strategy, which combined MeSH terms and entry terms, was finalized after three rounds of pre-retrieval. During these rounds, the search terms were adjusted based on their relevance, specificity, and comprehensiveness, ensuring they captured all relevant studies. We consulted with an information specialist to refine the search strategy and ensure its alignment with best practices.

The search terms used for both English and Chinese studies included repetitive transcranial magnetic stimulation (重复经颅磁刺激), stroke (脑卒中), cerebrovascular accident (脑血管意外), sleep (睡眠), sleep disorder (睡眠障碍), dyssomnias (睡眠异常), and insomnia (失眠). The Boolean search structure used across databases was as follows: (stroke OR cerebrovascular accident OR apoplexy) AND (sleep OR insomnia OR dyssomnias) AND (transcranial magnetic stimulation OR repetitive transcranial magnetic stimulation OR rTMS).

Taking Pubmed as an example, our literature search formula is as follows:

(((cerebral hemorrhage[Title/Abstract]) OR (((((stroke[Title/Abstract]) OR (cerebrovascular accident[Title/Abstract])) OR (apoplexy[Title/Abstract])) OR (cerebral in farction[Title/Abstract]))) AND (((sleep[Title/Abstract]) OR (insomnia[Title/Abstract])) OR (dyssomnias[Title/Abstract]))) AND (((transcranial magnetic stimulation[Title/Abstract]) OR (repetitive transcranial magnetic stimulation[Title/Abstract])) OR (rTMS[Title/Abstract]))).

This research has been registered with the PROSPERO International Systematic Review Registration Platform, and no deviations from the registered protocol occurred during the study. Full details of the protocol are available under Registration No. CRD42024589437, which can be accessed at https://www.crd.york.ac.uk/PROSPERO. The review adheres to the PRISMA 2020 guidelines (24), and the risk of bias was evaluated in accordance with the PRISMA extensions for network meta-analysis.

### 2.2 Selection variables

The Pittsburgh sleep quality index (PSQI) is a self-reported questionnaire used to measure sleep quality and disturbances over a one-month period. It has been validated in post-stroke populations, confirming its reliability and suitability for use in this cohort (25). The Pittsburgh Sleep Quality Index (PSQI) is composed of multiple components, with a total of 19 items distributed across seven domains, providing a global score to evaluate overall sleep quality. Each domain reflects a distinct aspect of sleep quality, including subjective sleep quality, sleep latency, sleep duration, sleep efficiency, sleep disturbances, use of sleep medication, and daytime fatigue, performance, or alertness. Scoring: Each component is scored on a scale from 0 to 3, with a global score ranging from 0 to 21. Higher scores indicate poorer sleep quality, with a global score above 5 typically suggesting significant sleep problems; however, this cut-off may vary depending on the clinical context and population norms (26).

While the PSQI provides valuable subjective insights into sleep quality, the inclusion of sleep efficiency, an objective measure derived from polysomnography, allows for a more comprehensive assessment of sleep quality, capturing both subjective experiences and objective sleep metrics. Sleep efficiency refers to the percentage of time spent asleep relative to the total time spent in bed (27). Polysomnography provides an accurate assessment of sleep efficiency by measuring the time spent asleep in comparison to the total time in bed. It is calculated as the total sleep time divided by the time in bed, multiplied by 100. Higher sleep efficiency indicates better sleep quality, as more time in bed is spent sleeping rather than awake (28). However, it is important to note that sleep efficiency derived from polysomnography can differ significantly from the self-reported estimates obtained from the PSQI. Polysomnography provides an objective, direct measure of sleep duration and disturbances, while the PSQI relies on subjective reports from the patient, which may lead to discrepancies between the two measures due to individual perceptions of sleep quality. Therefore, PSQI and sleep efficiency have been included in the meta-analysis to represent the impact on sleep quality following rTMS, providing a comprehensive assessment of both subjective and objective measures.

The Hamilton Depression Scale (HAMD-17) is a clinician-administered scale used to assess the severity of depressive symptoms. The 17-item version is preferred due to its wide use in clinical settings, particularly for stroke populations (29). It contains 17 items that measure various aspects of depression, including mood, guilt, insomnia, anxiety, and somatic symptoms. Each item is scored on a scale of 0 to 4 for symptoms such as depressive mood, guilt, suicidal tendencies, work and interest, slow thinking and speech, agitation, mental anxiety, somatic anxiety, and hypochondriasis. For symptoms like insomnia, shallow sleep, early awakening, gastrointestinal symptoms, general symptoms, sexual symptoms, weight loss, and insight, the scoring scale ranges from 0 to 2. The total possible score ranges from 0 to 52. Higher scores indicate more severe depression. A HAMD-17 score of 8 or above indicates the presence of depression, with severity categorized as follows: 0–7 (normal), 8–16 (mild), 17–23 (moderate), and ≥24 (severe) (30). Therefore, the HAMD-17 assesses the severity of depressive symptoms and serves as a tool for monitoring mood changes in post-stroke patients.

### 2.3 Selection criteria

We selected randomized controlled trials of the application of TMS in post-stroke sleep disorders patients. To be eligible for inclusion, studies needed to satisfy the following conditions: (1) The study subjects were patients with sleep disorders after stroke, all were aged>18 years, and all had stable vital signs.; (2) Diagnostic criteria for sleep disorders post-stroke were based on the International Classification of Sleep Disorders (ICSD-3) or The Diagnostic and Statistical Manual of Mental Disorders Fourth Edition (DSM-V), depending on the study.; (3) All participants had an MMSE score greater than 27, ensuring appropriate cognitive function.; (4)The experimental group received rTMS treatment with detailed standardized reporting, including coil type, stimulation protocol, stimulation intensity expressed as a percentage of the resting motor threshold, and other relevant parameters.; (5) In the control group, participants received conventional therapy (defined as a combination of pharmacological, behavioral, and/or physiotherapy interventions depending on the study), sham rTMS (no stimulation), conventional therapy combined with sham rTMS, or other treatments such as medication or acupuncture.; (6) outcomes comprising valid tests and measures of Pittsburgh Sleep Quality Index (PSQI), Sleep Efficiency, and Hamilton Depression Rating Scale-17 (HAMD-17); and (7) reporting the number of participants and all essential data for effect size calculations. Studies were excluded if they reported results from a previously published source (duplicated data). Our study protocol adhered to the Quality of Reporting of Meta-analyses (QUOROM) guidelines (31) and the Preferred Reporting Items for Systematic Reviews and Meta-Analyses (PRISMA) statements (24). All included studies were analyzed exclusively using the data reported in the published articles. The study adhered to the PRISMA checklist, ensuring transparency in the search strategy and the assessment of risk of bias, with the full checklist available in the Supplementary materials. The exclusion criteria are detailed in the figure.

### 2.4 Screening and data extraction

We extracted information on participants and trial characteristics using a structured form (Table 1) to ensure consistency, enable cross-study comparison, and reduce extraction bias. Specifically, this included participants’ mean age, sex, and baseline symptom severity, as well as trial characteristics such as first author, publication date, type of intervention, type of control, outcome assessment, and intervention duration.

**Table 1:** Characteristics of TMS parameters included in the study.

Studies and year	rTMS parameters				Comparison measure	Duration(wk)	outcomes
	Stimulation locations	Stimulation frequency(Hz)	Stimulation intensity	rTMS frequency			
Dong et al. (34)	r-DLPFC	1	80%MT	30 min × 1session × 7d/wk	M	3	PSQI, SE
Hua et al. (35)	r-DLPFC	1	80-120%RMT	15 min × 1session × 5d/wk	M	3	PSQI, SE, SRSS
Zheng et al. (36)	r-DLPFC	1	unclear	1session × 7d/wk	M	4	PSQI
Xu et al. (37)	r- DLPFC	1	80% ~ 120%MT	20 min × 1session × 6d/wk	M	4	PSQI, NIHSS
Wang et al. (38)	r- DLPFC	1	100%RMT	20 min × 1session × 6d/wk	A	4	PSQI, SE, HAMA
Chen et al. (39)	l-DLPFC	10	90%RMT	20 min × 1session × 5d/wk	SS + M	4	HAMD-17, SE
Ding et al. (40)	b-DLPFC	1	80%RMT	1session × 7d/wk	SS + M	2	PSQI, SE, BDNF
He et al. (25)	l-DLPFC	5	80%RMT	28 min × 1session × 5d/wk	M	8	HAMD-17, PSQI, CSS, MBI
Ma et al. (42)	l-DLPFC	5	90%RMT	28 min × 1session × 5d/wk	SS + M	4	HAMD-17, PSQI, SDS
Qi et al. (43)	r- DLPFC	1	80-120%RMT	20 min × 1session × 5d/wk	M	4	PSQI, NIHSS, SDS, SAS
Cao et al. (44)	l-DLPFC	0.5	unclear	1session × 5d/wk	M	6	SE, MMSE, MoCA, MBI, HAMD, BDNF
Li et al. (45)	r- DLPFC	1	80%RMT	1session × 5d/wk	M	6	PSQI, HAMA, HAMD-17
Yanget al (46).	l-DLPFC	1	90%RMT	20 min × 1session × 7d/wk	A	4	PSQI, HAMA, HAMD-17, GABA, 5-HT
Ma et al. (47)	r- DLPFC	1	80%RMT	10 min × 1session × 5d/wk	M	3	SAS, SE, SRSS
Hu et al. (48)	l-DLPFC	5	90%RMT	20 min × 1session × 5d/wk	A	4	HAMD-17, PSQI

C: control, E: experiment. Stimulation locations: r-DLPFC: right dorsolateral prefrontal cortex, l-DLPFC: left dorsolateral prefrontal cortex, b-DLPFC: bilateral DLPFC. Stimulation intensity: RMT: resting motor threshold, MT: motor threshold. Comparison measure: M: Medication, A: Acupuncture, SS: sham stimulation. Outcomes: PSQI: Pittsburgh sleep quality index, Sleep efficiency: SE, SRSS: self- rating scale of sleep, NIHSS: National Institute of Health stroke scale, HAMA: Hamilton Anxiety Scale, BDNF: brain-derived neurotrophic factor, CSS: chinese stroke scale, MBI: Modified Barthel Index, SDS: Self-rating depression scale, SAS: Self-rating Anxiety Scale, MMSE: Mini-Mental State Examination, MoCA: Montreal Cognitive Assessment, GABA: γ-aminobutyric acid, 5-HT: serotonin, SRSS: Self- Rating Scale of Sleep.

### 2.5 Risk of bias

Two researchers (CF and WZ) assessed the risk of bias in the included studies using the risk of bias assessment tool for RCTs as outlined in the Cochrane Handbook (24). The identity and role of researchers are clearly stated, and independent assessments and cross-validation were performed to ensure high inter-rater reliability. The evaluation criteria included the following domains: random sequence generation; allocation concealment; blinding of participants and personnel; blinding of outcome assessors; completeness of outcome data; selective reporting; and other potential sources of bias. Each domain was rated as ‘low risk’, ‘high risk’, or ‘unclear risk’ of bias based on the guidelines of the Cochrane Handbook (24), which provide specific criteria for judgment. To ensure reliability, two reviewers independently performed the risk of bias assessment, and any disagreements were resolved through discussion or consultation with a third reviewer.

Publication bias was evaluated using a comparison-adjusted funnel plot and Egger’s test. In the comparison-adjusted funnel plot, study-specific effect sizes were plotted against their standard errors while adjusting for different comparisons within the treatment network, under the assumption of transitivity and consistency across the network (23). Egger’s test was applied to assess funnel plot asymmetry, with a *p*-value <0.05 indicating potential small-study effects or publication bias (32). This approach accounts for the multi-arm structure of the treatment network and incorporates heterogeneity across studies.

### 2.6 Data synthesis and analysis

A frequentist network meta-analysis (NMA) using a random-effects model was employed to synthesize both direct and indirect evidence concurrently. All analyses were conducted using Stata statistical software version 16.0 and Review Manager software version 5.3 (provided in the Supplementary materials) (23). The effect size for continuous variables was assessed using mean difference (MD) with a 95% confidence interval (CI). CIs were computed using normal approximations from random-effects models, with between-study variance (τ^2^) estimated via restricted maximum likelihood (REML), under the assumption of approximately normal sampling distributions of study-level effect estimates. In cases where a closed loop existed, statistical inconsistency between direct and indirect evidence was evaluated through local (node-splitting method) and global (design-by-treatment interaction model) approaches (32). If the *p*-value exceeded 0.05, it indicated no statistically significant inconsistency, and the consistency model was applied for further analysis. A network plot was generated to illustrate the relationships among the different interventions, where the size of the nodes represented the sample size of each intervention, and the line thickness indicated the number of randomized controlled trials (RCTs) with direct comparisons. The SUCRA probabilities were calculated to assess the efficacy of various treatment strategies, with higher SUCRA values indicating a more favorable outcome (33). Cumulative rank-probability curves were derived by summing rank probabilities across categories, with surface under the cumulative ranking curve (SUCRA) corresponding to the normalized area under these cumulative curves.

## 3 Results of the network meta-analysis

### 3.1 Characteristics of the included studies

**Figure 1:**
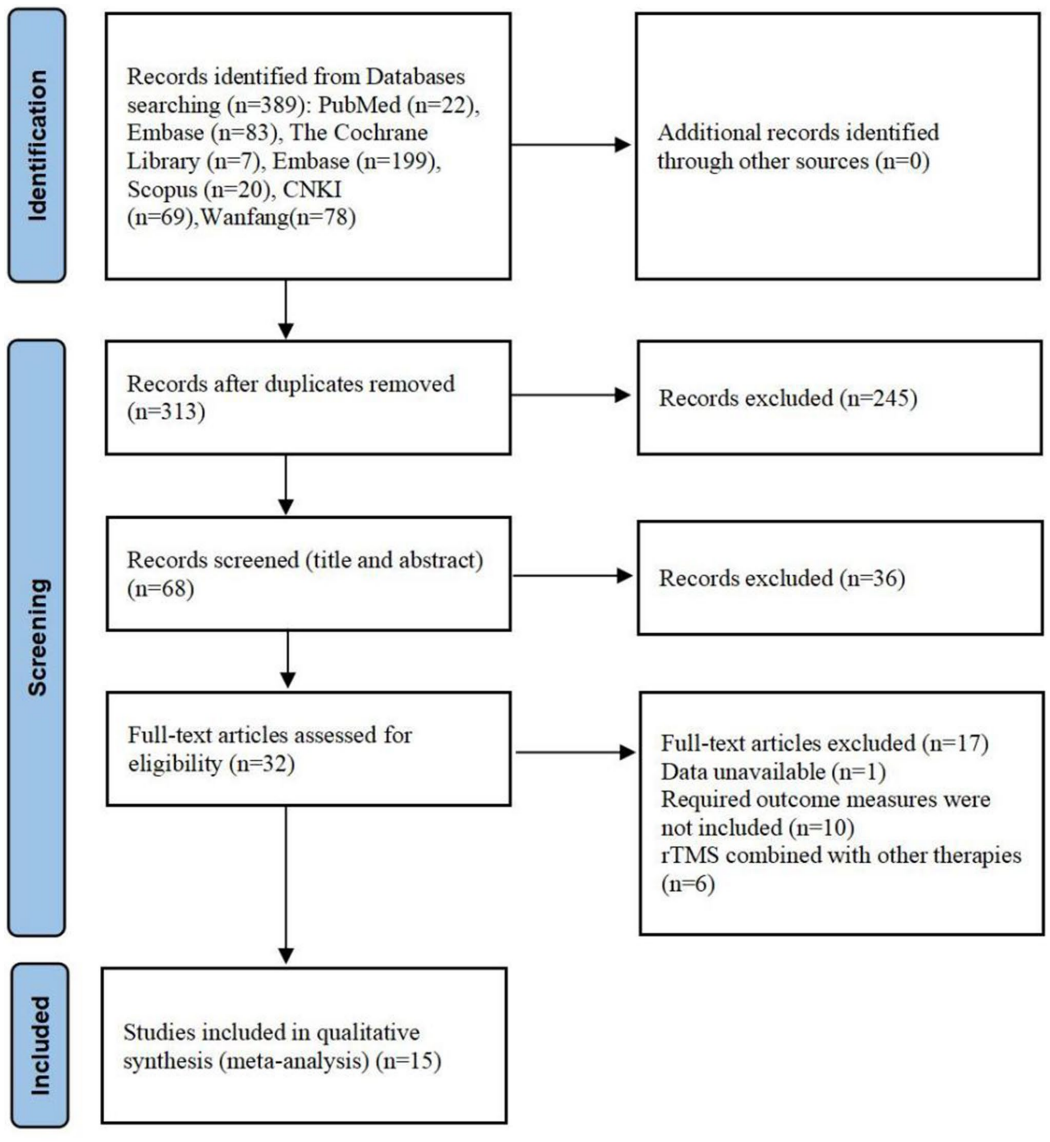
Screening process for the study’s literature selection.

Seven databases were searched utilizing the designated search strategy, yielding 389 papers during the initial screening. Among them, seven papers were retrieved from Cochrane Library, 22 from PubMed, 20 from Scopus, 199 from Embase, 63 from China National Knowledge Infrastructure, and 78 from the Wanfang Data databases. After removing duplicates, reviewing the titles, abstracts, and full texts, and conducting a quality assessment, 15 papers (34–48) were ultimately included. The detailed screening process, including the reasons for excluding the papers, is presented in Figure 1. Furthermore, the main characteristics of the included studies are summarized in Tables 2, 1.

**Table 2:** Basic characteristics of the included studies.

Studies and year	Diagnostic criteria		Age (years)		Duration (d/w/m)		Sample (n)	
	Stroke	Sleep disorders	C	T	C	T	C	T
Dong et al. (34)	①⑧	CCMD-3	56.72 ± 11.08	55.43 ± 10.49	16.87d ± 6.07d	17.52d ± 5.42d	50	50
Hua et al. (35)	①④	CCMD	60.33 ± 9.35	61.02 ± 9.81	33.96 ± 7.84d	35.40 ± 9.63d	45	45
Zheng et al. (36)	⑤	CDTI	57.51 ± 4.81	58.10 ± 5.10	8.93 ± 3.02w	9.04 ± 2.86w	50	50
Xu et al. (37)	①④	CCMD	64.2 ± 5.9	65.7 ± 6.1	37.6 ± 3.2d	39.4 ± 3.5d	28	30
Wang et al. (38)	①	CCMD-3	55.9 ± 7.1	59.6 ± 9.5	3.5 ± 2.7 m	3.8 ± 2.2 m	30	30
Chen et al. (39)	①④	CCMD-3	65.16 ± 9.18	64.06 ± 6.82	unclear		31	32
Ding et al. (40)	①④	PSQI	72 ± 4	70 ± 4	2.1 ± 0.5 m	2.2 ± 0.3 m	46	46
He et al. (25)	①④	PSQI	54.61 ± 9.81	58.52 ± 10.62	25.63 ± 6.81	24.75 ± 8.53	30	30
Ma et al. (42)	①	DSM-IV	56.9 ± 8.2	54.5 ± 8.3	15.0 ± 1.4d	14.9 ± 1.8d	20	20
Qi et al. (43)	①④	CDTI-2017	63.75 ± 5.92	63.12 ± 6.07	6.37 ± 1.09 m	6.44 ± 1.13 m	45	46
Cao et al. (44)	①③	PSQI	68.51 ± 3.87	68.76 ± 3.45	8.24 ± 1.67 m	8.43 ± 1.54 m	48	59
Li et al. (45)	⑥	PSQI	61.56 ± 7.60	60.53 ± 7.89	22.67 ± 4.96d	22.47 ± 4.64d	30	30
Yang et al. (46)	①	CDTI-2012	58.20 ± 5.03	58.10 ± 4.90	32.68 ± 15.53d	34.65 ± 15.58d	20	20
Ma et al. (47)	⑦	PSQI	58.41 ± 6.38	58.73 ± 7.29	9.15 ± 1.24 m	9.24 ± 1.39 m	50	50
Hu et al. (48)	⑥	Unclear	53 ± 10	50 ± 10	5.78 ± 3.04 m	5.32 ± 3.08 m	27	25

C control, E experiment. Diagnostic Standard for Stroke: ① CT/MRI; ② Chinese guidelines for diagnosis and treatment of acute ischemic stroke (version 2018); ③ China Expert Consensus on Clinical Practice of Post-stroke Depression; ④ Diagnostic criteria for cerebrovascular diseases in China (version 1995); ⑤ Guidelines for Early Rehabilitation and Treatment of Stroke in China; ⑥ Diagnostic essentials of various major cerebrovascular diseases in China (version 2019);⑦ Chinese guidelines for diagnosis and treatment of acute ischemic stroke (version 2014); ⑧ Chinese guidelines for cerebrovascular disease prevention and cure (version 2008). Diagnostic Standard for Sleep Disorders: PSQI=Pittsburgh sleep quality index; CCSSD=Chinese expert consensus on the assessment and management of stroke-related sleep disorders; CCMD-3 = Chinese classification and diagnostic criteria of mental disorders-third edition; CCMD=Chinese classification and diagnostic criteria of mental disorders; CDTI-2012 = Chinese guidelines for diagnosis and treatment of insomnia in adults (version 2012); CDTI-2017 = Chinese guidelines for diagnosis and treatment of insomnia in adults (version 2017); CCMD 2R = Chinese classification and diagnostic criteria of mental disorders-second revision edition; DSM-IV = The Diagnostic and Statistical Manual of Mental Disorders Fourth Edition.

A total of 15 randomized controlled trials (RCTs) involving 1,113 patients with PSSD were selected, with the patients having an average age range of 43–74.34 years. The frequency, target location, and intensity of rTMS differed across the included studies. Eleven studies employed low-frequency stimulation (≤1 Hz) (34–38, 40, 43–47), while four utilized high-frequency stimulation (≥5 Hz) (39, 41, 42, 48). The commonly targeted brain region was the DLPFC, with three localization methods: right, left, and bilateral. In particular, eight studies targeted the r-DLPFC (34–38, 43, 45, 47), six targeted the l-DLPFC (39, 41, 42, 44, 46, 48), and only one targeted the b-DLPFC (40). The stimulation intensity of rTMS is determined by the motor threshold of each patient. During the assessment of the motor threshold, patients are seated or placed in a supine position, and a single-pulse stimulation is applied to the motor cortex area controlling the thumb of the dominant hand. The motor threshold is established as the intensity at which thumb abduction is induced in 5 out of 10 stimulations (19). The motor threshold percentages varied across different studies. Five studies applied 80% of the motor threshold (34, 40, 41, 45, 47), four employed 90% (39, 42, 46, 48), three utilized an 80–120% range (35, 37, 43), and one used 100% (38). Only two studies did not specify the motor threshold (36, 44). The rTMS treatments were administered within a range of 2 to 8 weeks, with a frequency of once per day, 5–7 times per week, and a session duration ranging from 10 to 30 min (Table 1).

### 3.2 Risk of bias

**Figure 2:**
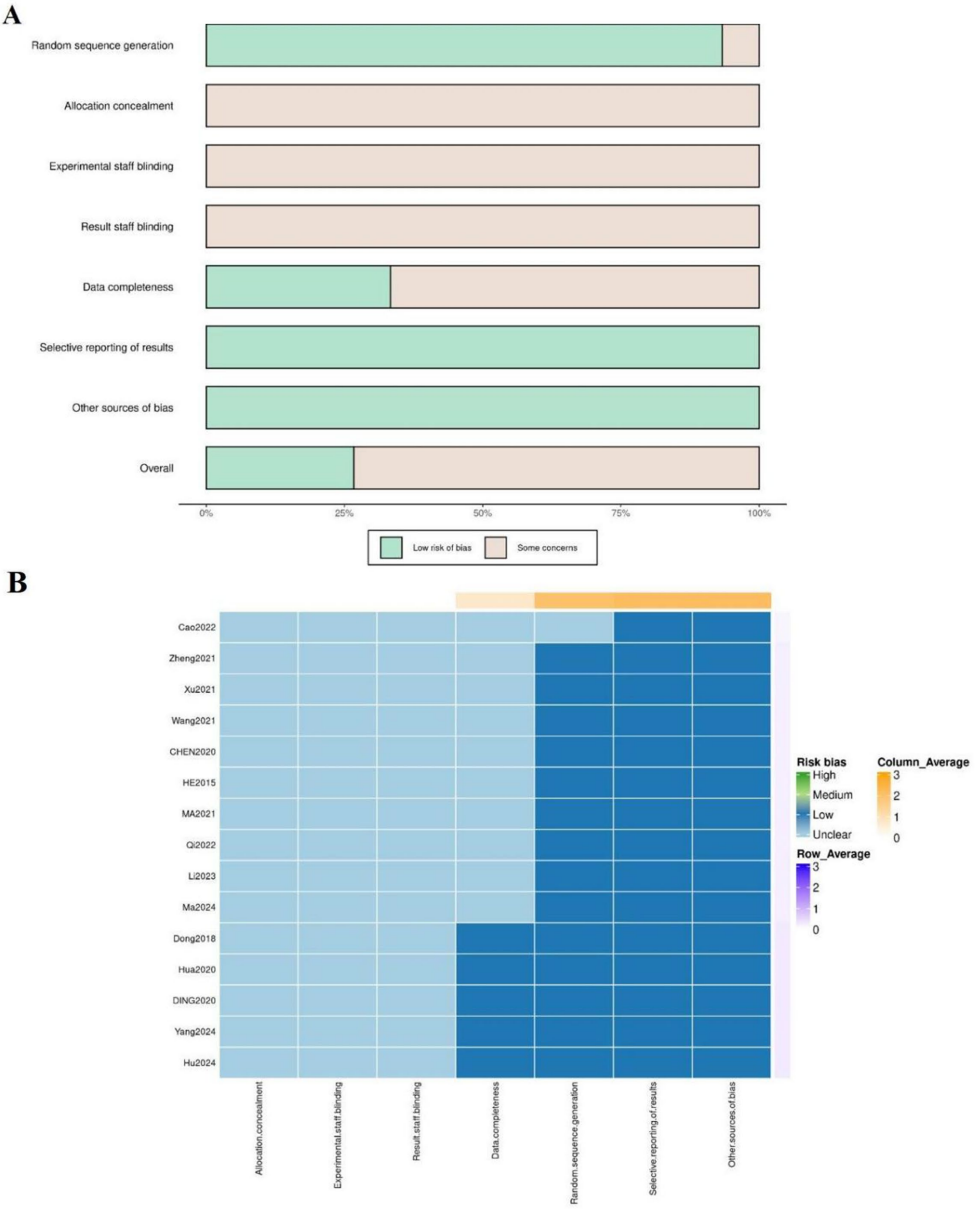
Assessment graph of risk-of-bias. B Summary of study quality and risk-of-bias. Except for 2 studies at high risk-of-bias for incomplete outcome data and selective reporting, respectively, others were rated as low or unclear.

Among the 15 included studies, 14 explicitly described the methods used for random sequence generation, such as computer-based randomization or random number tables; thus, they were assessed as having a low risk of bias for random sequence generation. However, the remaining study merely mentioned using randomization without providing details, leading to an unclear risk of bias for the randomization process. Furthermore, none of the studies provided information on allocation concealment or blinding of experimental staff. Consequently, all studies were considered to have an unclear risk of bias for allocation concealment and blinding of experimental staff. Moreover, all studies were evaluated as having a low risk of reporting bias. Patient dropouts were reported in five studies, indicating a low risk of bias for data completeness in those studies. Conversely, five other studies did not provide details on patient dropout, resulting in an unclear risk of bias for data completeness in those studies. Lastly, all studies were determined to have an unclear risk of bias for selective reporting of results and other sources of bias. The overall risk of bias for the included studies is illustrated in a risk-of-bias graph (Figure 2A) and a risk-of-bias summary (Figure 2B).

### 3.3 The results of the network meta-analysis

#### 3.3.1 PSQI scores

**Figure 3:**
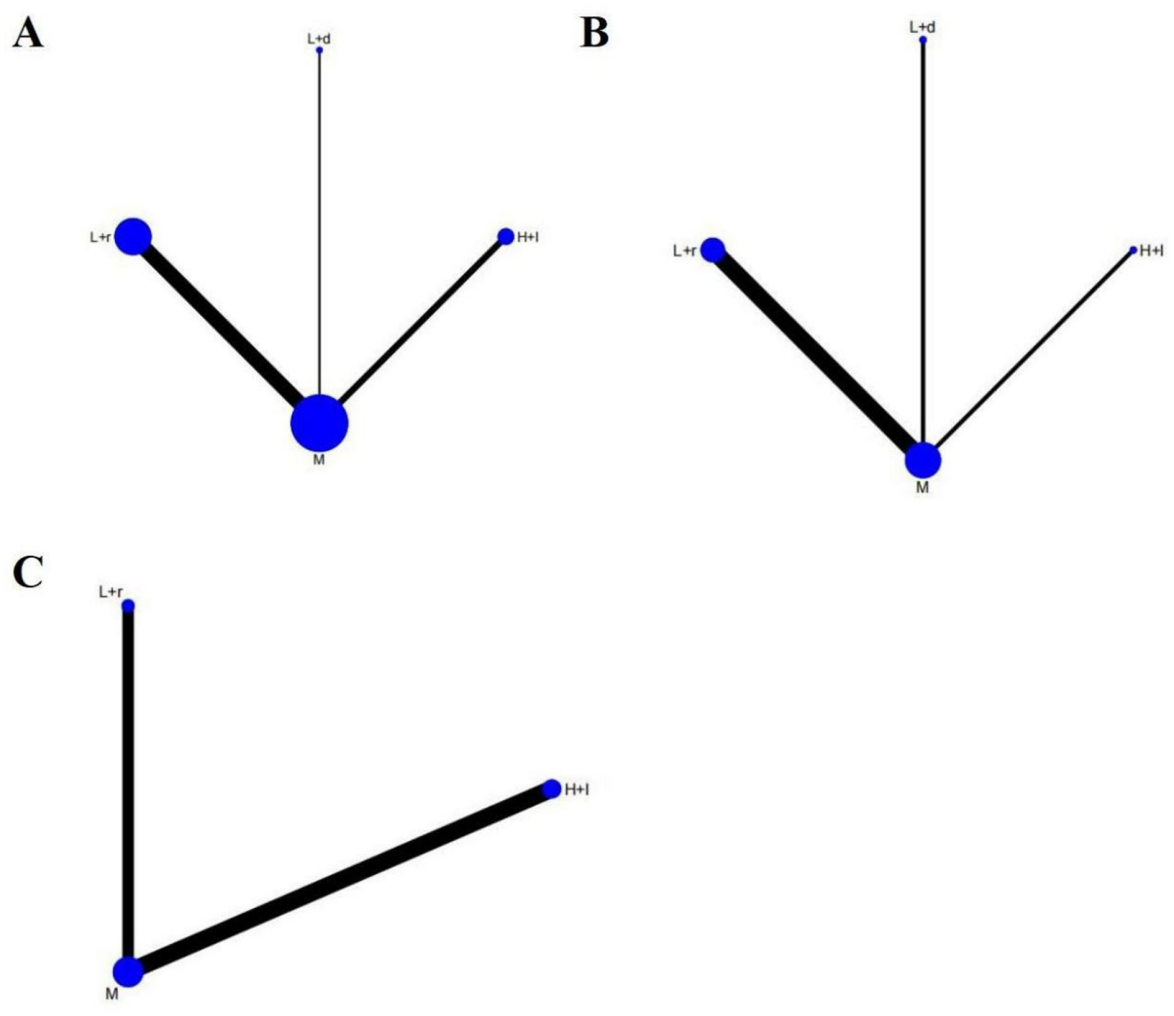
The network plots. **(A)** PSQI scores; **(B)** Sleep efficiency assessment; **(C)** HAMD-17 scores abbreviate: r-DLPFC: right dorsolateral prefrontal cortex, l-DLPFC: left dorsolateral prefrontal cortex, b-DLPFC: bilateral DLPFC, M: Medication.

**Figure 4:**
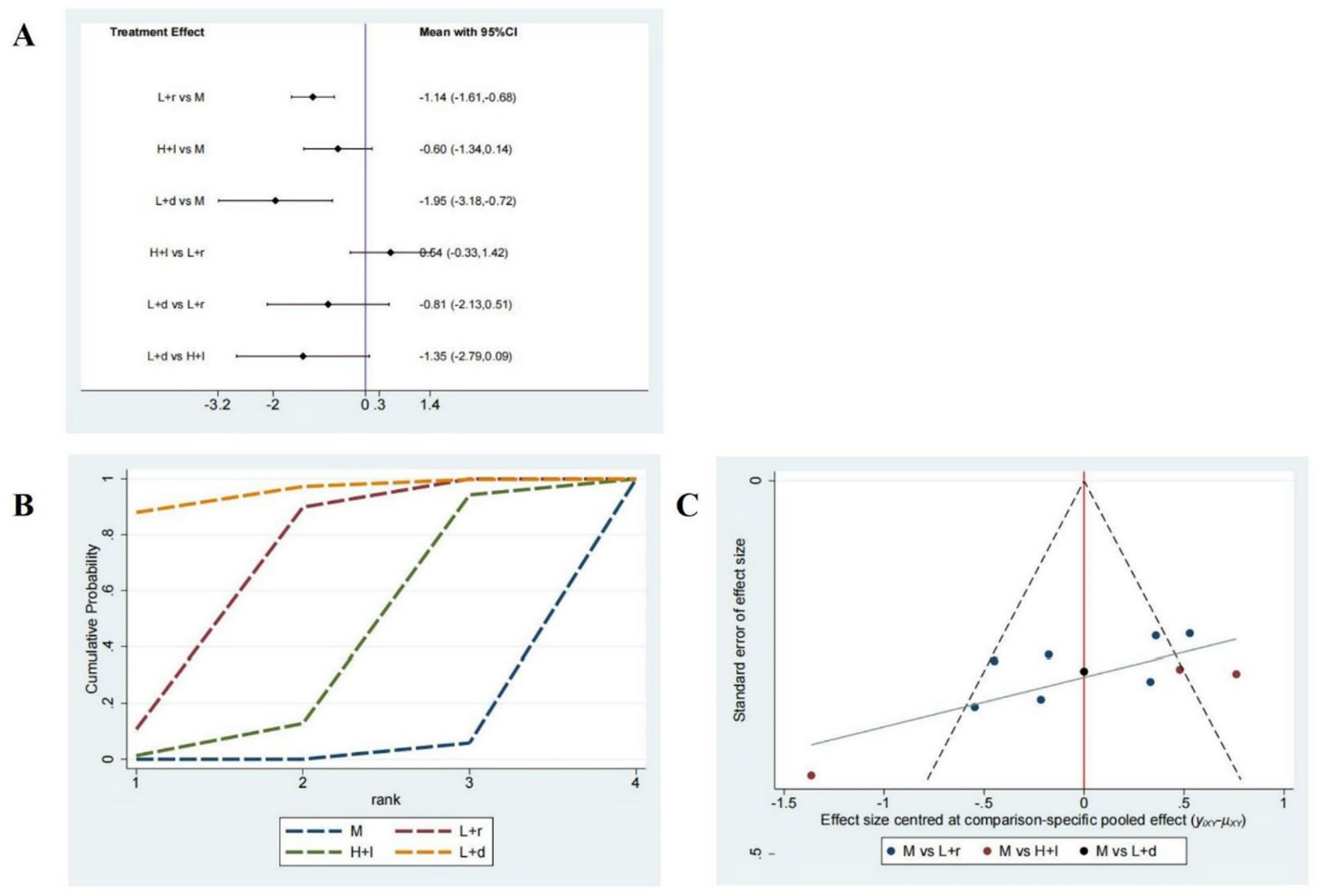
PSQI scores. **(A)** A forest map of pairwise comparison of PSQI scores; **(B)** Cumulative probability line chart of PSQI scores; **(C)** The funnel plots of PSQI scores abbreviate: r-DLPFC: right dorsolateral prefrontal cortex, l-DLPFC: left dorsolateral prefrontal cortex, d-DLPFC: dorsolateral prefrontal cortex, M: Medication.

Thirteen studies involving 906 patients reported on PSQI scores, and the network plot of these studies is presented in Figure 3A. As illustrated in Figure 4A, compared with pharmacotherapy alone, low-frequency rTMS targeting the r-DLPFC (SMD: −1.14; 95% CI: −1.61 to −0.68) and low-frequency rTMS targeting the b-DLPFC (SMD: −1.95; 95% CI: −3.18 to −0.72) led to significant differences in the PSQI score (*p* < 0.05). However, high-frequency rTMS targeting the l-DLPFC (SMD: −0.60; 95% CI: −1.34 to 0.14) did not result in a significant difference in the PSQI score compared with pharmacotherapy alone (*p* > 0.05). Moreover, no significant differences were detected among the different rTMS modalities (*p* > 0.05).

As depicted in Figure 4B, the surface under the cumulative ranking curve analysis (SUCRA) revealed the rankings of the different treatment methods according to their optimal probabilities as follows: low-frequency rTMS targeting the r-DLPFC (81.8%) > low-frequency rTMS targeting the b-DLPFC (73.8%) > high-frequency rTMS targeting the l-DLPFC (43%) > pharmacotherapy alone (1.3%). A symmetrical funnel plot presented in Figure 4C indicates no significant publication bias among these 13 studies.

#### 3.3.2 Sleep efficiency assessment

**Figure 5:**
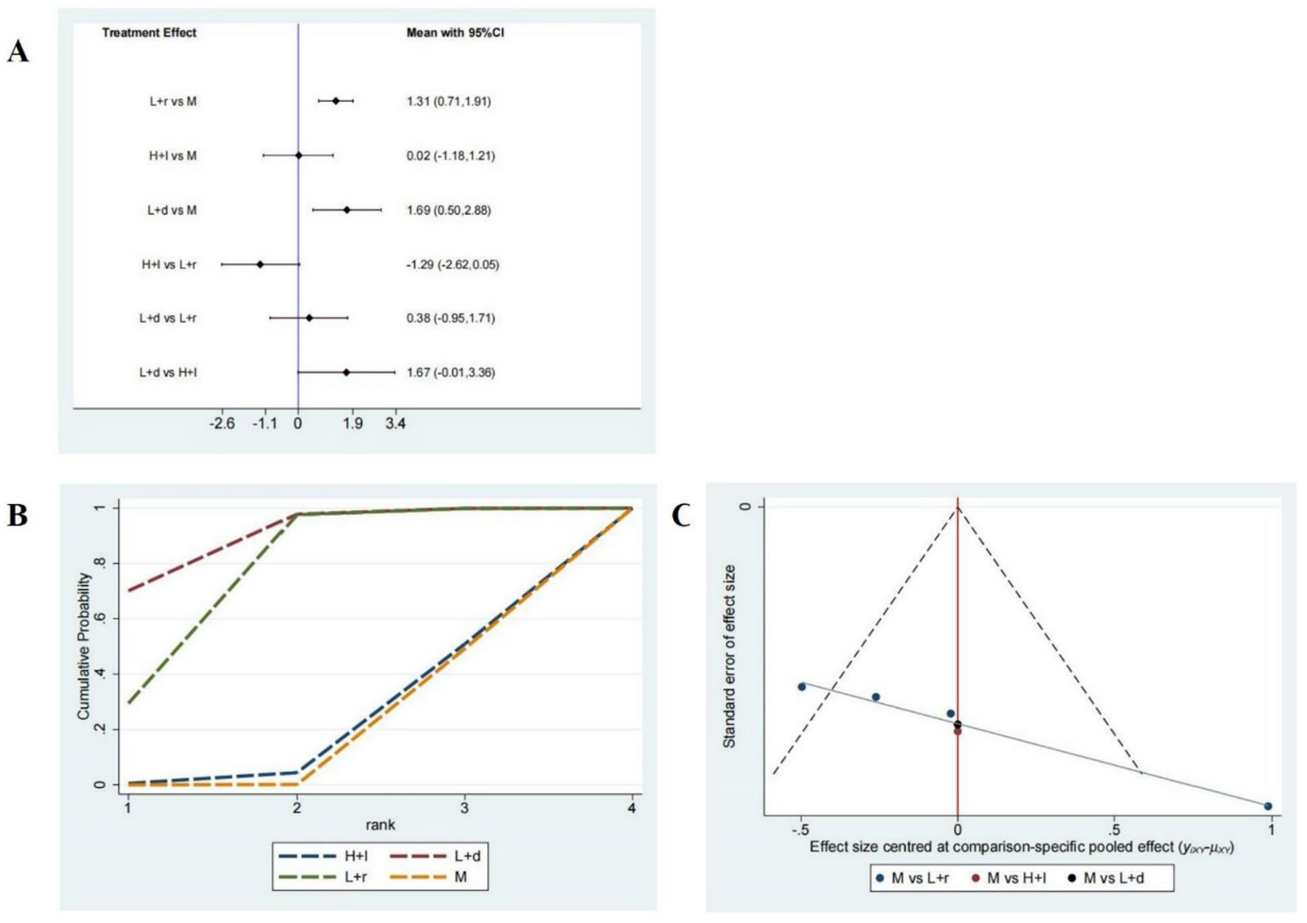
Sleep efficiency assessment. **(A)** A forest map of pairwise comparison of Sleep efficiency assessment; **(B)** Cumulative probability line chart of Sleep efficiency assessment; **(C)** The funnel plots of Sleep efficiency assessment abbreviate: r-DLPFC: right dorsolateral prefrontal cortex, l-DLPFC: left dorsolateral prefrontal cortex, d-DLPFC: dorsolateral prefrontal cortex, M: Medication.

Six studies comprising 512 patients examined sleep efficiency, and the network plot of these studies is shown in Figure 3B. As shown in Figure 5A, compared with pharmacotherapy alone, low-frequency rTMS targeting the r-DLPFC (SMD: 1.31; 95% CI: 0.71–1.91) and the b-DLPFC (SMD: 1.69; 95% CI: 0.50–2.88) yielded significant differences in sleep efficiency (*p* < 0.05). However, high-frequency rTMS targeting the l-DLPFC (SMD: 0.02; 95% CI: −1.18 to 1.21) did not produce a significant difference in sleep efficiency compared with pharmacotherapy alone (*p* > 0.05). Additionally, no significant differences in sleep efficiency were found among the different rTMS modalities (*p* > 0.05).

As presented in Figure 5B, the SUCRA analysis exhibited the rankings of the varied treatment techniques based on their optimal probabilities as follows: low-frequency rTMS targeting the b-DLPFC (98.9%) > low-frequency rTMS targeting the r-DLPFC (67.8%) > high-frequency rTMS targeting the l-DLPFC (17.4%) > pharmacotherapy alone (16%). A symmetrical funnel plot shown in Figure 5C reveals the potential presence of publication bias and small sample size effects.

#### 3.3.3 HAMD-17 scores

**Figure 6:**
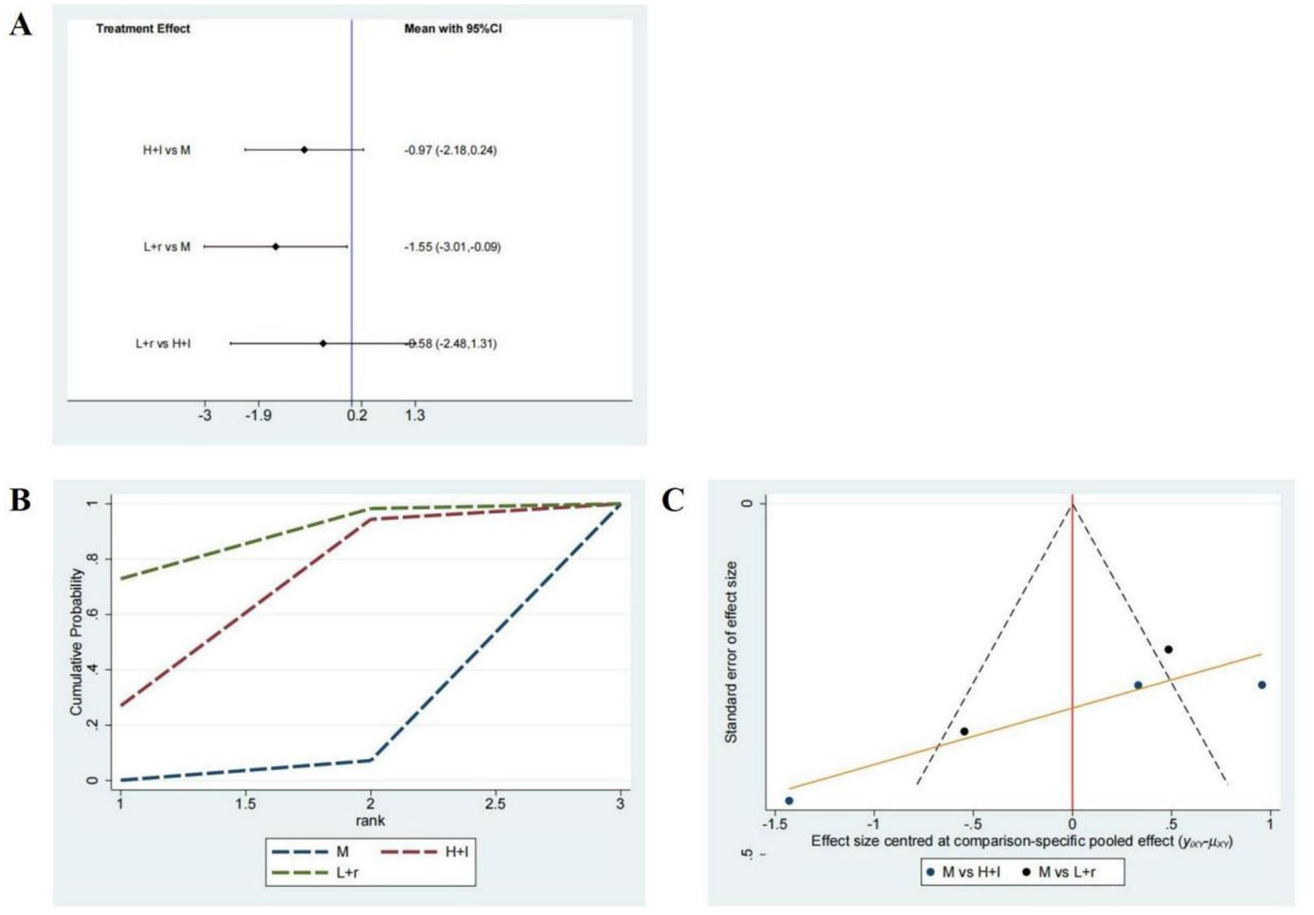
HAMD-17 scores. **(A)** A forest map of pairwise comparison of HAMD-17 scores; **(B)** Cumulative probability line chart of HAMD-17 scores; **(C)** The funnel plots of HAMD-17 scores abbreviate: r-DLPFC: right dorsolateral prefrontal cortex, l-DLPFC: left dorsolateral prefrontal cortex, d-DLPFC: dorsolateral prefrontal cortex, M: Medication.

Seven studies including 422 patients investigated the HAMD-17 scores, and the network plot of these studies is depicted in Figure 3C. As demonstrated in Figure 6A, compared with pharmacotherapy alone, low-frequency rTMS targeting the r-DLPFC (SMD: −1.55; 95% CI: −3.01 to −0.09) led to a significant difference in the HAMD-17 score (*p* < 0.05). In contrast, high-frequency rTMS targeting the l-DLPFC (SMD: −0.97; 95% CI: −2.18 to 0.24) did not result in a significant difference in the HAMD-17 score compared with pharmacotherapy alone (*p* > 0.05). Furthermore, no significant differences were observed between these two modalities (*p* > 0.05).

As shown in Figure 6B, the SUCRA analysis demonstrated the rankings of the various treatment methods according to their optimal probabilities as follows: low-frequency rTMS targeting the r-DLPFC (83.2%) > high-frequency rTMS targeting the l-DLPFC (62.7%) > pharmacotherapy alone (4.2%). A symmetrical funnel plot illustrated in Figure 6C shows the potential presence of publication bias and small sample size effects.

## 4 Discussion

Here, we systematically evaluated three rTMS modalities -low-frequency stimulation of the right DLPFC, low-frequency stimulation of the b-DLPFC, and high-frequency stimulation of the left DLPFC- or their efficacy in improving sleep function and alleviating depression in patients with PSSD. A total of 15 RCTs involving 1,113 patients with PSSD were included in the analysis. Our results revealed that low-frequency rTMS targeting the r-DLPFC and b-DLPFC significantly improved PSQI scores, sleep efficiency, and HAMD-17 scores. A forest plot of the pairwise comparisons revealed no statistically significant differences among the three rTMS modalities. The cumulative probability line chart indicated a rank-order preference for specific outcomes, consistent with the SUCRA values and rank probabilities shown in Figures 4B, 5B, 6B, and Supplementary materials. In particular, low-frequency rTMS of the r-DLPFC was optimal for enhancing PSQI scores, low-frequency b-DLPFC stimulation was most effective for augmenting sleep efficiency, and low-frequency r-DLPFC stimulation ranked highest for reducing HAMD-17 scores. The previous systematic review found that rTMS may have positive effects on sleep quality and mood in patients with PSSD (49). This aligns with our study in assessing the effectiveness of rTMS on PSSD. However, our study differs by incorporating a network meta-analysis to compare multiple rTMS modalities. Thereby Our findings provide additional comparative evidence on the relative efficacy of different rTMS modalities.

### 4.1 Understanding PSSD: prevalence, mechanisms, and impact

PSSD is a prevalent and critical barrier to recovery in stroke survivors, manifesting as abnormalities in the quality, quantity, or timing of sleep. Symptoms of PSSD commonly encompass nighttime awakenings, excessive daytime sleepiness, and disrupted sleep–wake rhythms (50), with stroke severity, age (51), and gender (52) serving as the contributing factors. Insomnia affects nearly 75% of stroke survivors, representing a significantly higher proportion than in the general population (25). Moreover, PSSD can exacerbate cognitive deficits, further impairing rehabilitation outcomes (53). The interplay between sleep disorders and depression after stroke is of major concern. PSSD often leads to fatigue, tension, and anxiety, which can compound the psychological burden, reduce the quality of life, and hinder recovery motivation of the affected patients (54). Poor sleep quality has been linked to greater depressive symptoms, and depression, in turn, is associated with worsened sleep disturbances, suggesting a bidirectional relationship between the two conditions after stroke (55). Along with psychological effects, PSSD exerts detrimental effects on the nervous and cardiovascular systems, thereby increasing the risk of recurrent cerebrovascular events and even mortality (56).

### 4.2 Neurophysiological insights into the mechanisms of PSSD

Although the stroke-induced effects on brain tissue are well-documented, the specific mechanisms underlying PSSD remain unclear. Furthermore, stroke-related changes in the upper airway anatomy and function may contribute to sleep-disordered breathing, a primary cause of these sleep disturbances (50). Previous research suggests that the disruption of the cerebellar neural circuits coordinating the upper airway and diaphragm can diminish the function of the upper airway muscles, ultimately contributing to sleep apnea (57). Moreover, damage to the brainstem nuclei involved in sleep–wake regulation may contribute to sleep disorders following a stroke. For example, stroke lesions in the hypothalamus have been associated with sleep disorders primarily characterized by reduced non-rapid eye movement (non-REM) sleep (58). In line with this finding, studies using animal models of middle cerebral artery occlusion have demonstrated a significant reduction in non-REM sleep duration, along with elevated levels of orexin and orexin receptor 1 (59). Orexin neurons in the hypothalamus play a crucial role in sleep inhibition, particularly during wakefulness (60). Apart from lesion location, post-stroke sleep-disordered breathing is also affected by several other factors. Studies have revealed higher rates of sleep-disordered breathing following strokes in the brainstem or bilateral hemispheres (61, 62). Conversely, Stahl (63) have found that the incidence and severity of obstructive sleep apnea syndrome following stroke were not significantly correlated with specific lesion locations. The pathogenesis of sleep disorders can also involve psychological factors. For instance, external environmental changes and psychological stress, such as those arising from stroke, may exacerbate sleep problems. In certain individuals, this worsening of sleep issues can lead to the progression from transient insomnia to persistent sleep disorders, particularly among those who are unable to regulate the stress caused by their condition. Our findings showed that Low-frequency rTMS targeting the right and bilateral DLPFC not only significantly improved sleep function in patients with stroke but may also alleviate depression.

Sleep is vital in the processes of neural repair and regeneration. Experimental studies have demonstrated that sleep deprivation reduces the proliferation of neurons and vascular cells and inhibits axonal growth, ultimately impeding neuroplasticity and recovery. This result highlights that sleep disturbances can significantly affect the restoration of neurological function (64). However, in clinical settings, neuroplasticity outcomes may also be affected by factors such as rehabilitation intensity, comorbidities, and individual variability. Moreover, sleep disorders can affect the brain’s autoregulatory mechanisms of cerebral blood flow, thus increasing the susceptibility of the brain to hypoxic damage. This oxidative stress environment also activates inflammatory pathways, leading to the release of inflammatory cytokines and further exacerbating neuronal injury (65, 66).

### 4.3 rTMS in PSSD: mechanisms and clinical implications

rTMS is an innovative, non-invasive neuroregulation technique. This stimulation method involves placing a coil perpendicular to the brain surface and passing an electric current through the coil to generate a pulsed magnetic field. Based on electromagnetic induction principles, this magnetic field induces a reverse current in the cortical tissue, which depolarizes local neurons, modulates cortical excitability, and influences neurotransmitter activity within targeted neural circuits. Such repeated, continuous, and rhythmic stimulations result in the modulation of the neural networks, eventually facilitating neuroplasticity (67). rTMS may treat PSSD through the following mechanisms:

1. Synaptic Plasticity: Synaptic plasticity, the activity-dependent modulation of synaptic efficacy, is a key mechanism by which rTMS may promote neural recovery. In stroke model rats, rTMS of the primary motor cortex reduced neuronal degeneration and synaptic loss, as shown by electrophysiological recordings and histological analyses (68). It also increased dendritic density and branching, supported by molecular markers such as dendritic spine morphology and BDNF expression (69).
2. Neurogenesis Modulation: rTMS has also been suggested to influence neurogenesis. After stroke, endogenous neural stem cells can contribute to the formation of neuroglial scars that impede neuronal regeneration, a phenomenon demonstrated primarily in preclinical studies (70). The application of rTMS in the infarcted side of the M1 cortex in ischemic stroke rats has been demonstrated to activate the expression of stromal cell-derived factor 1-alpha and the CXC chemokine receptor 4 axis in the perilesional cortex, which promotes neuronal regeneration (71). rTMS has also been reported to induce the neural differentiation of human embryonic stem cells in the forebrain (72).
3. Neuroinflammation Regulation: rTMS has been shown to regulate inflammation-related cytokines, thereby alleviating neuroinflammation. Neuroinflammation is a critical mechanism in the brain’s response to injury and disease. After a stroke, the damaged brain regions trigger acute and chronic neuroinflammatory responses, causing secondary cell death (73). Furthermore, preclinical studies suggest that long-term neuroinflammation can impair the proliferation and differentiation of neural stem cells, thereby limiting neurogenesis (74). Prior studies have revealed that rTMS can reduce the overexpression of pro-inflammatory cytokines such as IL-1β, IL-6, and TNF-*α*, as well as modulate the activation of glutamate receptors (including mGluR5 and NMDAR2B) and the proliferation of reactive microglia and astrocytes (75). For example, a study using a mouse model that underwent 3 h of ischemia found that rTMS applied to the infarcted side for 5 consecutive days led to a significant reduction in the expression of cytokines associated with inflammatory response and decreased microglial activation (76). All these mechanisms of rTMS highlight its potential as a therapeutic tool not only for improving neuroplasticity and neurogenesis but also for diminishing the harmful effects of neuroinflammation, thus contributing to PSSD treatment and neurological recovery.

In clinical practice, the effectiveness of rTMS largely depends on the combination of various stimulation parameters, including frequency, intensity, pulse number, duration, and the relative positioning of the coil to the target brain area. The DLPFC is the common target for rTMS, given its crucial role in generating and regulating emotions and its close connections to other emotion-related brain regions, such as the limbic system, amygdala, and cingulate cortex. Consequently, the modulation of DLPFC activity by rTMS not only directly influences DLPFC excitability but also indirectly affects these emotional control areas (77). For example, stimulating the DLPFC was demonstrated to reduce amygdala hyperactivity, thereby alleviating anxiety and negative emotions. Stimulation frequency is defined as the rate at which rTMS pulses are delivered. Low-frequency rTMS (1–5 Hz) is typically utilized to inhibit cortical activity, whereas high-frequency rTMS (10–20 Hz) is employed to activate cortical regions. The interhemispheric competition model, a theoretical model developed to explain rTMS applications in stroke rehabilitation, suggests that the balance of mutual inhibition between the two hemispheres via the corpus callosum is disrupted by stroke (78). Moreover, activity in the l-DLPFC is usually associated with positive emotions, while the r-DLPFC is more closely linked to negative emotions. Previous studies have indicated that depressive states are often associated with reduced l-DLPFC activity and a relative elevation in r-DLPFC activity (79). Hence, rTMS is frequently employed to activate the l-DLPFC (80) or inhibit the r-DLPFC (81), aiming to rebalance the activity in these regions and alleviate conditions such as insomnia. Lastly, research has also suggested that stimulating the r-DLPFC may result in comparatively fewer side effects, underscoring its better suitability as a target region in patients with lower tolerance to stimulation (81).

### 4.4 Implications and future directions

Our findings suggest that low-frequency rTMS targeting the r-DLPFC may be more effective than high-frequency rTMS of the l-DLPFC in improving sleep function and depression in patients with PSSD. Earlier studies have shown that different rTMS protocols are more suitable for specific symptoms of sleep disorders. For instance, right-side low-frequency rTMS appears to be more beneficial for patients experiencing difficulty falling asleep (82), while high-frequency stimulation of the l-DLPFC is more efficient in addressing the symptoms associated with early awakening and vivid dreaming (83). Additionally, the differences in rTMS efficacy may be related to individual variations in neural network structures, physiological characteristics, and symptom heterogeneity. In light of this aspect, some studies have recommended using neuro-navigation systems to select personalized stimulation targets, including a study that showed that 19 out of 27 patients with major depressive and generalized anxiety disorders had target sites in the L8Av region of the DLPFC (84). However, the clinical feasibility of implementing such personalized approaches in standard stroke rehabilitation settings presents several challenges. A nationwide survey of 1,129 physiatrists in South Korea revealed that while 86.1% expressed interest in utilizing neuro-navigation systems for rTMS therapy, only 7.4% reported having access to such systems within their institutions (85). The primary barriers identified included high device costs, lack of reimbursement coverage, and the need for specialized training. In contrast, the three localization methods mentioned in this study, based on standard brain localization, are more cost-effective, allowing for broader application in rTMS therapy.

Moreover, the process of systematically optimizing target selection and stimulation parameters requires further exploration. Although some patients may experience a sleep disorders relapse following rTMS treatment, scarce systematic research and supporting data are available on strategies for extending the therapeutic effects through long-term maintenance or personalized adjustment to stimulation frequencies. All these considerations underline the need for future studies to refine the rTMS therapeutic approach for PSSD, focusing on individualized treatment plans and sustained efficacy.

## 5 Limitations

Our study has several limitations that should be considered. First, the small subgroup sample sizes and lack of analysis based on stroke type (hemorrhagic vs. ischemic) and lesion location limit our ability to assess their impact on rTMS efficacy. Future research should address these factors to better understand the differential effects of rTMS in various stroke populations. Second, the studies in our analysis utilized varied durations of rTMS interventions. However, given the small sample sizes in each study, we could not conduct a subgroup analysis according to the intervention duration. Therefore, the potential impact of different rTMS intervention cycles was not fully considered in our analysis. Third, we were unable to formally evaluate network connectivity, sparsity, and the transitivity assumption due to the limited number of included trials and the relatively simple network structure. These aspects should be addressed in future studies with larger and more comprehensive treatment networks. Finally, some outcome measures were evaluated in a relatively limited number of studies, which might have reduced the reliability of the evidence for those specific outcomes.

## 6 Conclusion

This systematic review and network meta-analysis reveals that low-frequency rTMS targeting the r-DLPFC has an overall favorable effect on improving sleep function and alleviating depression in patients with PSSD. However, considering the limitations of this study, the efficacy ranking results should be utilized as a reference for clinical applications. Additionally, future high-quality RCTs are required to compare the effects and long-term efficacy of rTMS for sleep disorders with varied symptoms and/or severities.

## Data Availability

The datasets presented in this study can be found in online repositories. The names of the repository/repositories and accession number(s) can be found in the article/Supplementary material.
